# Carbon-Based Fluorescent Nano-Biosensors for the Detection of Cell-Free Circulating MicroRNAs

**DOI:** 10.3390/bios13020226

**Published:** 2023-02-04

**Authors:** Pooja Ratre, Nazim Nazeer, Roshani Kumari, Suresh Thareja, Bulbul Jain, Rajnarayan Tiwari, Arunika Kamthan, Rupesh K. Srivastava, Pradyumna Kumar Mishra

**Affiliations:** 1 Department of Molecular Biology, ICMR-National Institute for Research in Environmental Health, Bhopal 462030, India; 2 Department of Pharmaceutical Sciences and Natural Products, Central University of Punjab, Bathinda 151401, India; 3 Department of Biotechnology, All India Institute of Medical Sciences, New Delhi 110029, India

**Keywords:** nanosensor, circulating nucleic acids, biomarkers, point-of-care test, translational research

## Abstract

Currently, non-communicable diseases (NCDs) have emerged as potential risks for humans due to adopting a sedentary lifestyle and inaccurate diagnoses. The early detection of NCDs using point-of-care technologies significantly decreases the burden and will be poised to transform clinical intervention and healthcare provision. An imbalance in the levels of circulating cell-free microRNAs (ccf-miRNA) has manifested in NCDs, which are passively released into the bloodstream or actively produced from cells, improving the efficacy of disease screening and providing enormous sensing potential. The effective sensing of ccf-miRNA continues to be a significant technical challenge, even though sophisticated equipment is needed to analyze readouts and expression patterns. Nanomaterials have come to light as a potential solution as they provide significant advantages over other widely used diagnostic techniques to measure miRNAs. Particularly, CNDs-based fluorescence nano-biosensors are of great interest. Owing to the excellent fluorescence characteristics of CNDs, developing such sensors for ccf-microRNAs has been much more accessible. Here, we have critically examined recent advancements in fluorescence-based CNDs biosensors, including tools and techniques used for manufacturing these biosensors. Green synthesis methods for scaling up high-quality, fluorescent CNDs from a natural source are discussed. The various surface modifications that help attach biomolecules to CNDs utilizing covalent conjugation techniques for multiple applications, including self-assembly, sensing, and imaging, are analyzed. The current review will be of particular interest to researchers interested in fluorescence-based biosensors, materials chemistry, nanomedicine, and related fields, as we focus on CNDs-based nano-biosensors for ccf-miRNAs detection applications in the medical field.

## 1. Introduction

The development of non-invasive diagnostic methods, such as liquid biopsy, represents a significant advancement for people on the route to treating non-communicable diseases, including cancers. The significant benefits of liquid biopsy are that these tests are non-invasive, quick, accurate, and notably real time. Circulating tumor cells (CTC), DNA, and circulating tumor RNA are the central focus of emerging liquid biopsy tests. Liquid biopsy offers a wide range of potential applications in the detection and treatment of cancer, including screening for early detection. Because of their quick reaction times and enhanced analyte-discovering signals even in limited concentrations, carbon-based biosensors are currently attracting a lot of scientifi attention. Semiconductor quantum dots (QDs) were a modern form of nanostructure that demonstrated excellent qualities for diagnosis and therapy [1]. Controlling QDs size and distribution made it simple to adjust their electrical and optical characteristics. Yet, since certain semiconductor QDs include hazardous substances such as, cadmium, arsenic, selenium, and mercury, they have several disadvantages. One such disadvantage is cytotoxicity [2]. As a result, these QDs are neither environmentally friendly nor biodegradable. On the other hand, since their inception in 2004, carbon nanodots (CNDs) have been recognized as a strong contender to replace the extremely dangerous metallic semiconductor class of quantum dots [3]. This is partly because the characteristics of carbon quantum dots are widely acknowledged to include their nanoscale size, roughly flat or spherical morphologies, great water solubility, broad absorption in the UV-visible light spectrum, and vibrant fluorescence [4]. CNDs have an amorphous or nanocrystalline center, mainly sp^2^ carbon, graphite grid spacing, and outside oxygenic functional groups, allowing for water solubility and subsequent complexation. [5]. Graphene, graphene oxide, fullerenes, diamond nanoparticles, carbon quantum dots, and carbon nanotubes are derivatives of carbonaceous nanomaterials which have been broadly used in sensing applications. As the current form of carbon nanomaterial, CNDs is motivating considerable research efforts. Recent years have seen a rise in the popularity of CNDs with zero dimensions as eco-friendly, biocompatible, and inexpensive materials whose properties have sparked interest in a variety of applications [6].

These are organic molecular nanocrystals made up of several tiny sp^2^ carbon nanodomains with a sp^3^-hybridized carbon backbone [7]. They feature several intriguing and advantageous characteristics, including great biocompatibility, biological selectivity, minimal toxicity, and a stronger quantum size impact, and they are produced utilizing affordable manufacturing procedures. CNDs play a crucial role in disease-related small biomolecule analysis through the creation of diverse nanoplatforms, which have the benefits of being simple, cheap, and quick to detect. Specific circulating markers can be detected using fluorescence, colorimetric, electrochemical [8], photoelectrochemical (PEC), and electrochemiluminescence (ECL) approaches [9]. The major factors contributing to the carbon nanodots’ huge achievement are its facile manufacturing and the comparatively established synthesis techniques using various physical or chemical techniques [10]. These methods are often classified as top–down or bottom–up procedures. Top–down ways include cutting large carbon-based components into nanosized fragments, whereas bottom–up approaches require synthesizing molecules that act as nanostructured precursors [11]. Green one-step techniques for creating CNDs have recently received a lot of emphasis due to their ease of use, ability to save time, lack of pollution, and low price [12]. Several plant species are used as renewable carbon sources in the production of green CNDs. The leaves, flowers or roots of a plant are the components that are utilized most frequently. According to reports, green-synthesized CNDs have quantum yield (QY) ranging from 3 to 75%, with a typical QY of 14% [13]. Their size can also be adjusted by adjusting the bandgap of the element used in synthesis. In contrast to the conventional techniques based on straight CNDs–analytes reactions, future work in green CNDs sensing can take these concepts to sensing analytes into consideration [14]. Owing to CNDs’ typically low cytotoxicity and resilience to photobleaching, sensing is one of the most researched uses of CNDs. Because of their advantageous photostability, photoluminescence, and charge transfer, CNDs are useful as optical sensor components. For biosensing applications, CNDs must be able to identify markers through precise couplings with target molecules (such as antibodies, aptamers, and enzymes) and modify their optical characteristics (or start catalysis) as a result of those interactions [15]. Biomarkers are frequently viewed as a type of quantitative tag that denotes certain biological states of human bodies amid these molecular analytes [16]. As a result, biomarker sensors have immense promise for illness early detection and individualized treatment. Small non-coding RNAs known as microRNAs (miRNAs) are thought to contribute significantly in the identification of disease signatures [17]. Furthermore, there is a strong correlation between disease progression and exosomal miRNA levels. Numerous techniques have been employed to date for the identification of miRNAs, notably Northern blot, quantitative real-time polymerase chain reaction (qRT-PCR), gene microarrays, electrochemistry and electrochemiluminescence. Such methods still have flaws, though, such as complicated maintenance and high expenses [18]. Because of their versatility, label-free monitoring, and extraordinary sensitivity, photonic biosensor technologies such as CNDs have generated a lot of attention [19]. Additionally, photonic biosensors may be multiplexed and made smaller, which meets the needs of point of care testing. As a result, label-free nanophotonic biosensors have gained attention as a viable solution for the creation of fresh methods for the detection of circulating miRNAs. Nanophotonic biosensors have proven their detection efficiency and clinical diagnostic capabilities [20,21]. For many nanophotonic applications, the ability to create novel nanomaterials with carefully crafted optical properties is of great interest [22]. These papers provided a thorough description of CNDs applications for miRNA detection, including everything from green production to surface functionalization techniques and physiochemical features.

## 2. Physicochemical Properties

### 2.1. Structural Properties 

CNDs have an amorphous nanostructure that is quasi-spherical in shape. According to the studies, the photoluminescence in CNDs is mostly caused by their surface characteristics, with lesser interaction from sp^2^ clusters (crystallinity) and the quantum confinement. As a result, the energy band gap of CNDs is influenced both by the quantum confinement phenomenon and the surface-active group. CNDs have the lattice constant which is halfway in between the range of a graphene or graphite lattice. CNDs are often intrinsically linked to surface passivation via derivatization or modifications. Several carboxyl groups impart excellent biocompatibility and water solubility to the CNDs surface, and various synthesis processes result in different chemical compositions for CNDs [23]. CNDs are carbon nanomaterials with or without a crystalline phase and have a sphere form [24]. Its layers are spaced around 0.34 nm apart, which is consistent with the crystalline graphite’s gap [25]. They have a system of linked or altered chemical organic compounds, such as oxygen- as well as amino-based groups, etc., on their top. They are basically hybridized with oxygen-containing functional groups and have an external sp^3^ configuration and an inside sp^2^ configuration [26].

### 2.2. Optical Properties

#### 2.2.1. Light Absorption of CNDs 

Generally, CNDs exhibit an evident absorption coefficient in the UV-visible spectrum. Regardless of what method they use to make, the majority of CNDs have an absorbance peak that ranges from 260 to 323 nm [27]. Absorption peaks in spectroscopic analysis may occasionally be caused by the n-p transition of C-O bonds or the p-p shift of C-C molecules. It is discovered that passivating the coating of CNDs with different compounds causes the absorbance to shift to higher wavelength [28], for instance. The absorption region was extended in Cl-doped CNDs, and the photo-excited particles were pushed to separate. Recently, enhanced CNDs with strong visible optical absorption and a near-IR optical absorption edge have been produced and are being used in the fields of bioimaging and sensing [29].

#### 2.2.2. Photoluminescence (PL)

One of the most intriguing optical properties of CNDs dots is its PL [30]. The principal components of luminescence are their intrinsic state and defect state emission. Whereas the precise process is uncertain, several factors have been connected to luminescence, including carbon excitons, emissive trapping, the quantum confinement impact, aromatic structures, oxygen-containing units, free zigzag domains and edge defects [31]. Most C-dots have a similar PL range mainly spanning the blue area (wavelengths between 400 and 500 nm). The dependence of the emission peak position on the excitation wavelength is an interesting feature of the PL of CNDs [32]. An intriguing aspect of the PL of CNDs is the dependency of the emission peak position on the excitation wavelength. which might be affected by surface reorganizations, electrical state alterations, and both core and external functional groups [33]. They differ from other nanomaterials, such as ordinary quantum dots, due to this peculiarity. The transition gap between the lowest unoccupied molecular orbital (LUMO) and the highest occupied molecular orbital (HOMO), which is inversely proportional to size, affects the size reliance of CNDs [34]. Additionally, 10 nm CNDs displayed better PL than 30–50 nm CNDs, which is reasonable considering that smaller CNDs have a higher surface area to volume ratio. Since it is dependent on the carbon center and the presence of an oxygen-functional group, it is easy to tune PL CNDs and may be beneficial for biosensing applications [35]. 

### 2.3. Chemical Properties

A typical description of CNDs is a carbogenic core with surface functional groups. The most prevalent elements are carbon and oxygen, which are coupled with several carboxylic acids [36]. To enhance the chemical properties of carbon nanodots, two main strategies include doping and surface functionalization. The top of CNDs may have a wide variety of oxygen-containing groups, such as epoxy/ether, carboxylic acid, carbonyl, and hydroxyl [37]. Furthermore, it is simple to dope additional elements, particularly N and S, into CNDs. In addition to being a crucial link between CNDs and intriguing biomedical applications, surface functionalization, or changing the functional groups on the surface of C-dots, is an effective technique for modifying the emission characteristics of C-dots [38]. It enables the production of a wide range of C-dots for sensing applications by altering their functional groups on the surface, which serve as receptors, thus establishing a solid association between C-dots and biological systems [39].

### 2.4. Photostability

**Figure 1:**
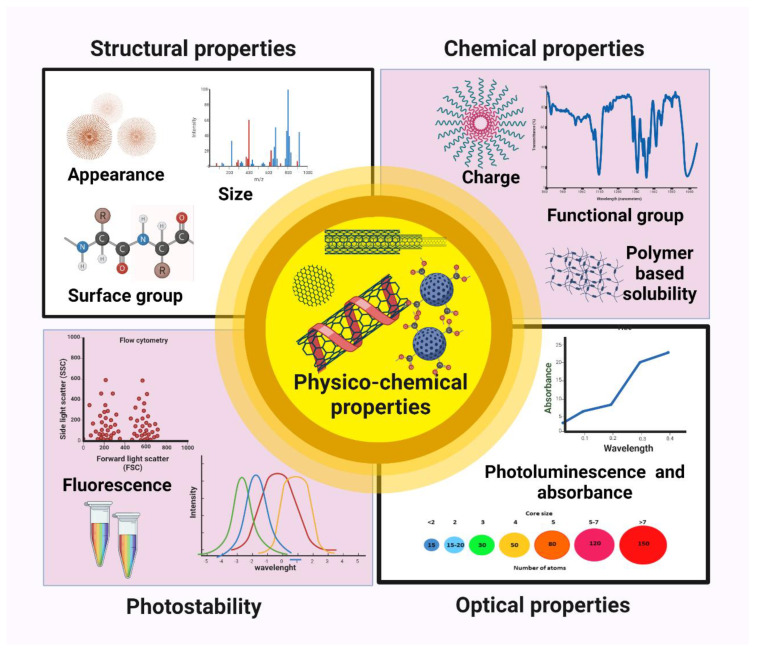
Physicochemical properties of CNDs.

For a prolonged period of sustained illumination, photostability indicates that the fluorescence emission brightness of tagged cells holds steady [40]. It is important to consider how well CNDs work as fluorescent probes for cell imaging by comparing their photostability in various cancerous and healthy cells [41]. CNDs feature lower toxicity and therefore possibly higher bioactivity than semiconducting QDs such as CdSe and CdS due to their improved photostability over flashing and photobleaching. The fluorescence of nitrogen- and boron-doped CNDs was strong and steady despite pH changes and exhibited great photostability. In addition, the excision of an oxygen group from CNDs might improve their photostability and reduce their cytotoxicity, providing molecular scale data to help with the development of far better and biocompatible CNDs. These all show that perhaps the CNDs are quite stable and would have high photostability for the modern sensing applications (Figure 1) [42].

## 3. Synthesis of CNDs

Both “bottom–up” and “top–down” strategies may be used to create semiconductors based on CNDs. Bottom–up strategies encompass thermal decomposition, electrochemical carbonization, microwave irradiation synthesis, and hydrothermal/solvothermal treatment. Top–down methods includes laser ablation, ultrasonication, arc discharge and electrochemical oxidation. For both these processes, a strict reaction environment is frequently needed, including high-grade carbon substrates, extreme heat, powerful alkali/acid solutions, and hazardous organic solvent [43].

### 3.1. Top–Down Approach

**Figure 2:**
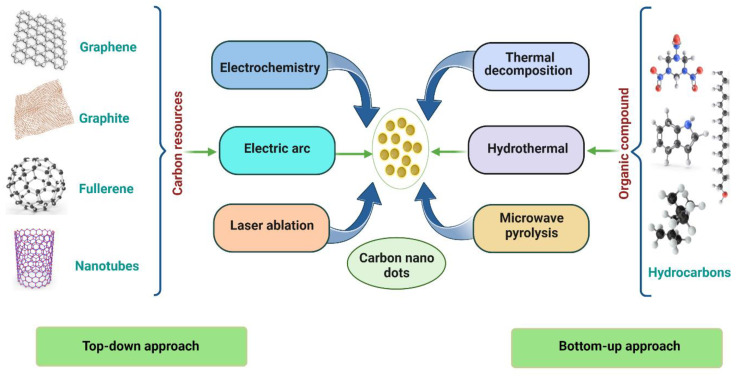
Top–down and bottom–up approaches for the synthesis of CNDs by using hydrothermal, microwave pyrolysis, thermal decomposition, laser ablation and other different synthesis methods.

Due to their simplified preparation procedures, top–down approaches are appropriate for the mass manufacturing of CNDs nanomaterials. The top–down approach “cuts” carbon particles including CNTs and graphite into CNDs via an arc discharge, laser ablation, or chemical oxidation [44]. Arc discharge and laser ablation are the most commonly used top–down methods for producing CNDs. Gonçalves et al. used laser ablation in water solution, N-acetyl-l-cysteine, and NH2-polyethylene glycol (PEG200) to create passivated CNDs [45]. Chemical oxidation involves introducing oxygen-containing hydrophilic functional groups into carbon nanostructure complexes by oxidizing them with a potent acid. The carbon nanostructures become water-soluble, which facilitates their discharge into the fluid [46]. Scientists have also produced CNDs by hydrothermally slicing graphene sheets. Wang et al. used graphene oxide as the precursor to create C-dots using the hydrothermal process with microwave assistance. Carbon dots that are hydrophilic, hydrophobic, or even amphiphilic can be made using microwave-assisted synthesis. A simple one-step microwave-assisted synthesis of hydrophobic C-dots was described by Mitra et al. Using glucose as a starting material, Ma et al. reported the ultrasonic synthesis of N-doped C-dots (Figure 2) [47].

### 3.2. Bottom–Up Approach 

In a “bottom–up” approach, CNDs are synthesized from small carbon molecules using microwave, hydrothermal, and pyrolysis methods. Basic principles involve burning and heating carbon precursors. CNDs can be prepared very efficiently through a bottom–up approach using a plethora of starting materials, and the choice of reactants determines their properties, especially in surface coating. Much more important is the fact that the roots of the carbon substrate can have a massive effect on the CNDs’ characteristics, including their sensing capabilities. Another advantage of the bottom–up approach is the easy addition of heteroatoms and other dopants. Sucrose, citric acid, amino acids, and food waste are carbon sources [48].

Direct pyrolysis, the pyrolytic technique, or the carbonization of precursor materials at high temperatures are standard methods for producing carbon dots. Zhu et al. were the first to employ microwave pyrolysis as a synthesis mode, using a dissolved saccharide and PEG-200. The size of CNDs increased with reaction time as this solution was heated in a 500 W microwave [49]. The yield of CNDs increases, and side reactions are reduced during microwave pyrolysis. Many CNDs variants have been created through direct thermal decomposition, in which precursors are heated in an inert environment until they are carbonized. Solvents are then used to extract them. The carbonization of small molecular precursors is used in the bottom–up synthesis of CND. One of the most common bottom–up synthesis approaches produces CND from a mixture of citric acid and a nitrogen-containing molecule such as urea [50]. When these molecular precursors are pyrolyzed by microwaves or in an autoclave, the synthesis readily produces a black nanopowder of CNDs, which is highly dispersible in water and displaying remarkable fluorescent properties. Depending on the conditions, these CNDs can display blue, green, or red emissions, although extensive purification is often needed to isolate CNDs from molecular intermediates produced during the synthesis. Bottom–up methods were efficient routes to produce fluorescent CNDs on a large scale. For example, small molecules and polymers can undergo dehydration and further carbonization to form CNDs [51]. 

### 3.3. Preparation of CNDs Using Green Approach

**Figure 3:**
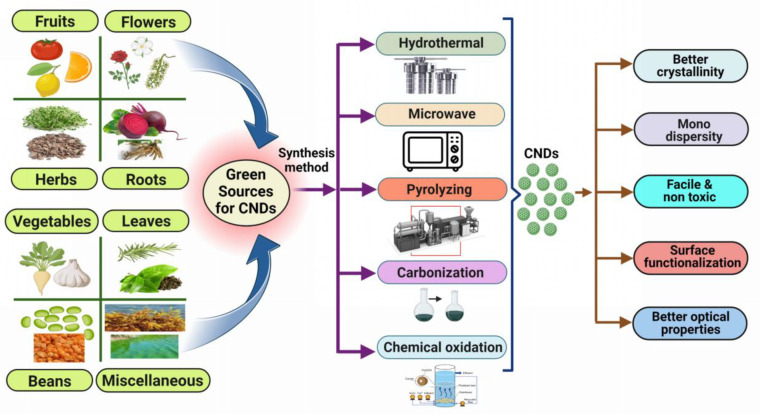
Various natural precursor for the synthesis of CNDs by using hydrothermal, microwave, pyrolysis chemical oxidation and carbonization as green approaches.

CNDs synthesized from biological sources play a significant role in biomedical and environmental applications, including bioimaging, biosensing, metal ions detection and electrocatalytic oxidations [52]. Green synthesis has attracted the interest of scientists because it is cost-effective, less hazardous, eco-friendly, less time-consuming, and requires lower temperatures (Table 1) [53]. The production of CNDs from mostly reusable substrates includes naturally available raw materials that are relatively cheap and simple to make. CNDs made from natural sources can be used to transform low-value biomass waste into rich and valuable products. The low manufacturing cost and constant availability of raw ingredients for CNDs synthesis have made it a viable procedure for the industry also. Additionally, no dangerous organic solvents are required; instead, an aqueous solution may be used, increasing the CNDs water solubility (Figure 3) [54].

Recently, Hashemi et al. manufactured CNDs using a low-cost, simple, and green one-step hydrothermal process, producing luminous CNDs with high quantum yield from red beetroot as an organic source. According to the paper, red beetroot was sliced into small pieces and mixed with deionized water, continuously swirling for 20 min before being sonicated for an hour. The mixture was then placed in a Teflon-lined autoclave and heated in the oven (180° for 10 h). It was then centrifuged (1000 rpm for 30 min) and filtered to obtain the CNDs solution. To obtain a pure CNDs solution, the mixture was dialyzed for three days to remove contaminants [55]. As a result, in the current context, the green synthesis approach of C-dots produces high C-dot yields at a cheap cost because of low-cost raw materials. The simple procedure adopted, as well as the fluorescence qualities found in C-dots derived from environmentally sourced materials, open the way for harmless and biocompatible C-dots to be used in sensing approaches. The study describes a single-step hydrothermal strategy to synthesize colored CNDs from maple leaves to specifically capture cesium ions. The CNDs made emit blue fluorescence and varied in size from 1 to 10 nm. Based on the electron transfer method, these CNDs were successfully employed in glycerol electro-oxidation catalysts and cesium-detecting probes [56]. Arumugham et al. made CNDs using catharanthus roseus (white) leaves as the carbon source without the addition of an oxidizing agent or an encapsulant. These CNDs have excellent antioxidant activity and bioimaging potential against MCF-7 cells as well as strong fluorescence (FL) emission, high water solubility, stability, and non-toxicity, among other properties [57].

**Table 1:** List of Various Natural Sources Used in the Preparation of Carbon Nanodots Using Different Green Synthesis Methods.

S. No.	Source	Method of Synthesis	Size	Percentage Yield	Detection Limit	Inference	References
1	Banana peel	Microwave treatment	5 to 15 nm	16.0%	1.82 × 10–17/mol	CNDs are fabricated by the microwave treatment of banana peels in a single pot for the determination of colitoxin DNA in human serum.	[58]
2	Sargassum fluitans	Hydrothermal	2–8 nm	18.2%	-	A hydrothermal method is used to produce CNDs from waste seaweed sargassum fluitans (*S. fluitans*) to detect DNA.	[59]
3	Tomato juice	Hydrothermal	1.3–3.7 nm	13.9%	0.3 ng/mL	CNDs are synthesized by hydrothermal treatment of tomato juice for the sensing of carcinoembryonic antigen.	[60]
4	Limes	Pyrolyzing	5-10 nm	-	-	The pyrolyzing process is used to synthesize CNDs for the detection of hepatitis B virus DNA.	[61]
5	Lemon juice	Carbonization	6–9 nm	-	0.23 mM	Carbonization of lemon juice is performed to form CNDs for the detection of l-tyrosine.	[62]
6	Lemon	Pyrolyzing	10 nm	-	0.0049 µM	Synthesis of CNDs from a lemon by the process of pyrolysis for the detection of doxorubicin hydrochloride in human plasma.	[63]
7	Syringa oblata lindl	Hydrothermal	1.0–5.0 nm	12.4%,	0.11 μM	A hydrothermal method is used to fabricate CNDs from syringa oblata lindl for sensors and cell imaging.	[64]
8	Grapefruit	Hydrothermal	>30 nm	20%	-	Grapefruit is used to create CNDs using a hydrothermal process for the detection of *E. coli bacteria.*	[65]
9	Alfalfa and garlic	Hydrothermal	1.3–6.9 nm	10%	86 nM	A hydrothermal method is used to form CNDs from alfalfa and garlic as a fluorescent probe for cysteine, glutathione, and homocysteine.	[66]
10	Catharanthus roseus (white flowering plant)	Hydrothermal carbonization	-	-	-	Catharanthus roseus (white flowering plant) is hydrothermally carbonized to create CNDs to detect the Al^3+^ and Fe^3+^ ions.	[57]
11	Lemon juice	Hydrothermal	-	-	-	The one-pot facile hydrothermal approach was used to create highly luminous carbon dots (C-dots) from lemon juice.	[67]
12	Daucus carota	Hydrothermal	-	7.60%	-	A hydrothermal method is used to produce CNDs from Daucus carota to detect mitomycin.	[68]
13	Natural polymer starch	Hydrothermal	2.25–3.50 nm	-	-	Hydrothermal treatment of natural polymer starch is performed to produce CNDs.	[69]
14	*P. acidus*	Hydrothermal	5 nm	12.5%	-	CNDs are produced by a hydrothermal process from *P. Acidus* for live cell imaging.	[70]
15	Citrus peel powder	Sand bath heat-assisted method	4.6 ± 0.28nm	-	-	The sand bath heat-assisted method is utilized to form CNDs from citrus peel powder for free radical scavenging and cell imaging.	[71]
16	Lentil	Hydrothermal	7 ± 4 µm	10%	3.0 µg	A hydrothermal method is used to form CNDs from lentils for the colorimetric determination of thioridazine hydrochloride.	[72]
17	Rose flowers	Hydrothermal	1.0–5.0 nm	-	0.02–10 µM	CNDs are produced by a hydrothermal process from rose flowers for the determination of diazinon.	[73]
18	Saffron	Hydrothermal	>20 nm	23.6%	1.8 n/mol	A hydrothermal method is used to produce CNDs from saffron for the sensing of prilocaine.	[74]
19	Valerian root	Hydrothermal	>10 nm	14%	0.6 ng/mL	Valerian root has been used to make CNDs using a hydrothermal process for the determination of imipramine.	[75]
20	Rosemary leaves	Hydrothermal	Approx. 5 nm.	18%	8 ng/mL	Rosemary leaves have been used to make CNDs using a hydrothermal process for the determination of thiabendazole in juices.	[76]
21	Beetroot	Microwave	5 & 8 nm	6% & 5%	-	CNDs made from aqueous beetroot extract by the process of a microwave for in vivo live animal imaging applications.	[77]
22	Eutrophic algal blooms	Chemical oxidation	Approx. 8 nm	13%	-	Eutrophic algal blooms have been used to make CNDs using chemical oxidation for in vitro imaging.	[78]
23	Green tea leaf	Pyrolyzation	2 nm	14.8%	-	Synthesis of CNDs from green tea leaf by the process of pyrolysis for the sensing of gefitinib.	[79]
24	Waste tea residue	Chemical oxidation	3.2 nm	2.47%	Be 0.04 μg /mL	Waste tea residue has been used to make CNDs using chemical oxidation for the quantification of tetracycline.	[80]
25	Palm shell powder	Chemical oxidation	4–10 nm	6.8%	0.079 µM	CNDs are synthesized by the chemical oxidation method from palm shell powder for the sensing of nitrophenol.	[81]
26	Soybeans	Ultrasonic-assisted method	2.4 nm	16.7%	0.9μM	An ultrasonic-assisted method is used to produce CNDs from soybeans to detect Fe^3+^ ions.	[82]

Abbreviations: nanometer (nm), millimeter (mm), micrometer (µm), carcinoembryonic antigen (CEA), *Escherichia coli* (*E. coli*), *Phyllanthus acidus* (*P. acidus*), nanograms (ng).

Kumar et al. simply heated orange juice at 120 °C for 150 min without using any specialized methods or chemicals. These spherical CNDs have a restricted size distribution, as seen by recorded electron microscopy. The hydrothermal technique used for this study is a reactive technique that produces CNDs of good yield and high quality [83]. In a recent article, Saleem et al. present a one-step flexible approach to produce fluorescent CNDs utilizing carrot root species. The synthesized CNDs worked as nano-vehicles for the mitomycin medication delivery. By breaking hydrogen bonds in the moderately acidic extracellular milieu of the tumor, the manufactured CNDs efficiently interacted with the mitomycin drug. This caused the release of the mitomycin [84].

As probes for the detection of heavy metal ions, fluorescent nitrogen-doped CNDs with 5.23% nitrogen content were made utilizing a one-pot microwave processing of lotus roots. The properties of egg yolk oil (EYO) were studied by Zhao et al., who utilized microscopy, spectrophotometry, and chromatography to detect the CNDs that were present in the EYO after it had been extracted and purified using water, dialysis, and ultrafiltration (EYO CNDs). The bleeding periods of mice treated with CNDs were noticeably shorter than those of control animals in tests on liver and tail hemorrhaging. According to coagulation tests, EYO CNDs stimulate and activate the fibrinogen system as well as the intrinsic blood coagulation system. Therefore, EYO CNDs has the capacity to stimulate hemostasis, which may prompt more research into this component of traditional Chinese medicine [85]. Xiao et al. present an inexpensive, easy, and effective microwave pyrolysis method to synthesize highly amino-functionalized fluorescent (CNDs). Through the dehydration of chitosan, the formation and functionalization of CNDs were successfully accomplished. Using a brand-new, quick microwave-assisted method that entails two stages, CNDs with an average size of 9 nm were created from an aqueous solution of raw cashew gum (RCG). A composite of partly depolymerized CG and CNDs was created at the end of the procedure [86].

In a study, the ecologically friendly one-step electrodeposition method for creating GR-based hybrids was employed which avoids chemically reducing graphene oxide (rGO), which would cause further pollution. The entire process is straightforward and takes only a few minutes. Combining the benefits of GR, CNTs, and CS, the GR/CNTs/CS hybrid was created and might be used to trap organophosphate pesticides [87]. In another study, a simple, cost-effective, and environmentally friendly method for producing ternary nanocomposites of carbon, polydopamine, and gold was demonstrated. The technique did not employ harsh reaction conditions such as those found in hydrothermal or high-temperature techniques. Excellent electrocatalytic activity was demonstrated by the CNTs/PDA/AuNPs modified electrode to oxidize chloramphenicol [88]. One more research study covered a synthesis of multiwall carbon nanotube/Cu_2_O-CuO ball-like composite (MWCNTs/Cu_2_O-CuO) adopting a green hydrothermal approach which had been investigated as a novel sorbent for the solid-phase extraction of uranium utilizing inductively coupled plasma mass spectrometry [89]. 

## 4. CNDs Surface Functionalization Using Various Chemistry

The absorbance and photoluminescence characteristics of the carbon dots are significantly influenced by surface functionalization and passivation through modifications to their capacity to interact with other organic compounds, ions, drugs, and living things [90]. The starting constituent and synthesis process also influence the degree of bioconjugation of CNDs, which affects their functional properties, such as biosensing and imaging. It is advantageous for therapeutic applications when functionalization methods leave CNDs surfaces with neutral or negative net charges [91]. As a result, proteins circulate in the blood for a longer period because negatively charged surface groups may hinder protein binding due to electrostatic repulsion. In contrast, neutral groups can evade immune system clearance. A crucial stage in the hydrodispersion of CNDs is the alteration of their surface. However, to provide a focused interaction between the fluorescent nanocrystals and the biological target, crosslinking with macromolecules is required. By affixing them to specific ligands such as vitamins, proteins, peptides, or antibodies, luminescent CNDs may be particularly useful for cellular-focused imaging. The functionalizing ligands could be coupled directly to the organically surface layer of the CNDs, or they could be linked via a substance such as polyethylene glycols, 2,2′-(methylenedioxy)-bis-ethylamine, nickel-nitrilotriacetic acid, or the biotin–streptavidin complex. It is important to remember that the innate activity of biological agents coupled to CNDs should be preserved during conjugation with biomolecules [92]. CNDs surfaces can contain a wide variety of oxygen-containing groups, including carbonyl, hydroxyl, carboxylic acid, and epoxy/ether, because of oxidation processes. In addition, components such as N and S can easily be doped into CNDs. Covalent conjugation between CNDs and biomolecules takes place in the reaction of one functional group with another, resulting in the formation of a covalent bond. Covalent linking among CNDs and molecules or other particles can be accomplished using different types of coupling agents [93].

### 4.1. Amine–Amine Coupling

Amine–amine coupling is a class of homo-bifunctional reaction in which the amine groups of two molecules bind to each other covalently using a dithiobis(succinimidylpropionate (DSP) as a linker. Each end of DSP has two distinct amine-reactive sites [94]. After being activated by N-Hydroxysulfo succinimide, CNDs bind in one, and at another end, amine-containing biomolecules bind. This coupling reaction can alternatively be carried out in two steps or in one step. All ingredients (biomolecules, CNDs, and DSP-coupling reagents) are combined together in the case of a single step. In a two-stage process, the DSP linker is used to first activate the amine group on CNDs, and then, in the second phase, the activated CNDs are combined with aminated biomolecules [95]. Other linkers used for conjugating aminated CNDs with amine group biomolecules are glutaraldehyde and aldehyde from the Schiff base [96].

### 4.2. Amine–Thiol Conjugation

Indirect crosslinking between the amines in CNDs and the thiols in biomolecules is the most popular method for producing nanoconjugates. Water-soluble sulfosuccinimidyl-4-(N617 maleimidomethyl) cyclohexane-1-carboxylate (sulfo-SMCC) as a linker forms a stable amide bond with the amine group of CNDs at one end and a thioether bond with the thiol group of the biomolecule at another end [97]. A nucleophilic reaction takes place between the sulfhydryl group and the maleimide double bond to form a thioether bond. Compounds of maleic anhydride are frequently employed as functional group bonds [98].

### 4.3. Histidine–Nickel Nitrilotriacetic Acid Conjugation

Fluorescent dyes, such as CNDs coupled to the Ni-NTA complex, have demonstrated the capacity to detect biological processes by selective binding to His-tagged biomolecules. The intriguing aspect of this interaction is that it remains unaffected by high salt, non-ionic detergent, or highly thermal decomposition environments [99]. The reaction mechanism involves the coating of CNDs by aminated polymer followed by its activation using sulfo-SMCC, which is a hetero-bifunctional crosslinker in borate buffer. In the next step, the NTA reacted with activated CNDs to obtain NTA-modified CNDs. The NTA-modified CNDs were treated to form the Ni complex by reacting with an excess of NiCl_2_·6H_2_O to obtain Ni–NTA modified QDs, which is highly specific for binding with his-tagged biomolecules [100].

### 4.4. Thiol–Maleimide Conjugation

The thiol–maleimide reaction is used to conjugate CNDs onto biomolecules via thiol conjugation. For functionalization on CNDs, there are amine to thiol sensitive cross-linkers. Sulfosuccinimidyl-4-(N-maleimido-methyl) cyclohexane-1-carboxylate (sSMCC) has an end NHS and a maleimide on another end. In this process, DNA that has been tagged with an amine first reacts with NHS to produce DNA maleimide [101]. The polymer-capped CNDs that have been reduced to exhibit thiols are then coupled to the DNA maleimide. Proteins and antibodies might be conjugated to CNDs using the same approach [95]. Alternately, a more traditional use of this strategy is attaching CNDs that exhibit primary amines to DNA that has undergone thiol modification [102].

### 4.5. Conjugation of Thiol with Amine Group by SPDP Linker

This is a another type of conjugation that links amine group of biomolecules to a thiol group of CNDs. Succimidyl 3-(2-pyridyldithio)propionate (SPDP) is a bifunctional linker utilized to functionalize amine-ending DNA to CNDs [103]. The linker has a cleavable pyridyl disulfide with one side and an NHS group on another side. In the first step, the functionalization of amine-DNA with the linker is performed using the linker. The disulfide bond is split in the second stage in the vicinity of a reductant to produce reactive thiols [104]. The reducing agent and the resulting pyridine-2 thione group are eliminated, and the DNA functionalized with a reactive thiol is combined with CNDs that have been maleimide-functionalized [105].

### 4.6. Antigen–Antibody Conjugation

Antibodies can be substantially specific, attaching to just a tiny segment (epitope) of an antigen, and they can distinguish between significantly similar epitopes. They detect proteins relying on both their structure and their content [106]. Antibodies conjugation with CNDs requires two basic chemistries, carbodiimide and site click [107]. During the site click linkage process, azide-alkyne cycloaddition mediator-adapted CNDs were attached to an azide-modified antibody. Similar to EDC-NHS mediated coupling, carboxyl groups of CNDs are activated for direct interaction with primary amines of antibody and nucleic acid via amide crosslinking in carbodiimide conjugation [108].

### 4.7. Conjugation of CNDs Containing Epoxide Functionality

**Figure 4:**
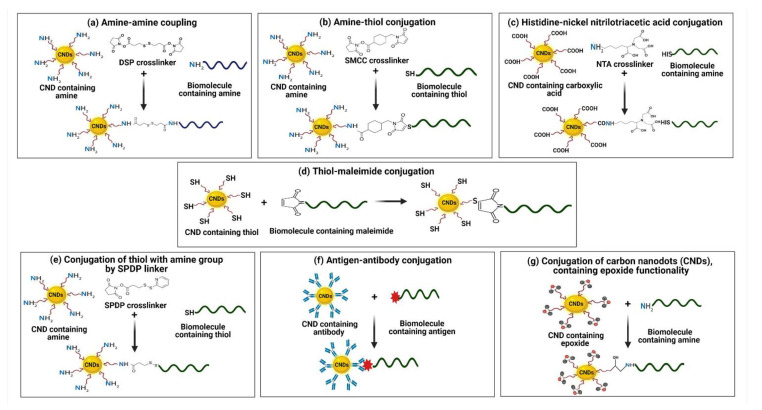
Conjugation of biomolecules with carbon nanodots via different chemistries and cross-linkers. (**a**) Amine–amine conjugation by using DSP crosslinker. (**b**) Conjugation via amine-thiol and SMCC as crosslinker. (**c**) Histidine–nickel nitrilotriacetic acid-based conjugation of biomolecules with CNDs. (**d**) Conjugation of CNDs with biomolecules by using thiol maleimide. (**e**) Conjugation of thiol containing biomolecules with CNDs by using SPDP crosslinker. (**f**) Antigen–antibody-based conjugation of CNDs with biomolecules. (**g**) Conjugation of CNDs containing epoxide functionality with biomolecules.

Mounting the epoxide group on the edge of CNDs can be highly beneficial since it interacts with various functional groups that are present on biomolecules. When amine, thiol, or hydroxyl group bearing molecules interact with CNDs that possess epoxide, secondary amine bonds, thioether bonds, and ether bonds are formed [109]. For instance, the fabrication of multiplex immunosorbent assays for the identification of three mycotoxins makes use of the synthesis of silica-based CNDs that are linked with epoxide group. An epoxide ring-opening reaction with an amines group of biomolecules (antibodies and nucleic acids) was used to conjugate epoxy-terminated Si-CNDs (Figure 4) [110].

## 5. Cell-Free Circulating MiRNAs

Small non-coding RNAs, or miRNAs, are a subclass that includes short 18–27 nucleotide sequences that function as essential gene regulators by inhibiting translation or interfering with RNA degradation [111]. Non-coding nucleotide sequences have an impact on a variety of vital cellular processes, including cell differentiation, proliferation, growth, motility, and apoptosis, as well as the development of diseases. These molecules may serve as potential biomarker candidates for the diagnosis and prognosis of various illnesses. Several studies have found that some miRNAs are significantly altered during development and disease, including cancer, heart disease, diabetes, nervous system, renal, and liver diseases. It is worth noting that miRNA expression is a dynamic process, and cell-free nucleic acid measurement has the potential to be a cost-effective, non-invasive technique for disease screening and prognostic assessment [112]. miRNAs are typically found in the cellular microenvironment; according to studies, however, a significant fraction of miRNAs, known as cell-free circulating miRNAs (ccf-miRNAs) or extracellular miRNAs, are in the extracellular milieu. These circulating miRNAs were eventually discovered in blood plasma, serum, saliva, urine, and other bodily fluids. However, depending on a person’s health and the disease’s cause, the concentration of miRNAs may change significantly. These molecular entities can represent current pathophysiological conditions and are thought to be derived from blood cells, circulating tumor cells, or other disease-affected tissue cells [113].

### 5.1. MiRNAs Biogenesis

Non-coding RNAs are molecules that do not translate into proteins (ncRNAs). Most of the human genomes are translated into non-coding RNAs, according to recent discoveries (ncRNAs) [114]. About 30% of miRNA genes are found in intergenic regions or the antisense orientation of genes, each having its promoter and regulatory units. Numerous miRNAs are found in exons and introns. The precursor-miRNA is produced in the nucleus after the transcribed miRNA (80 nucleotides) is processed to form the pri-miRNA stem-loop structure (pre-miRNA). After a series of enzymatic processes and transfer from the nucleus to the cytoplasm, the mature miRNA is then created. These mature miRNAs are loaded onto the AGO and combined to form the effector miRNA-induced silencing complex C (miRISC). Either the RISC complex deteriorates, or the passenger strand is loaded with the complex [115].

**Figure 5:**
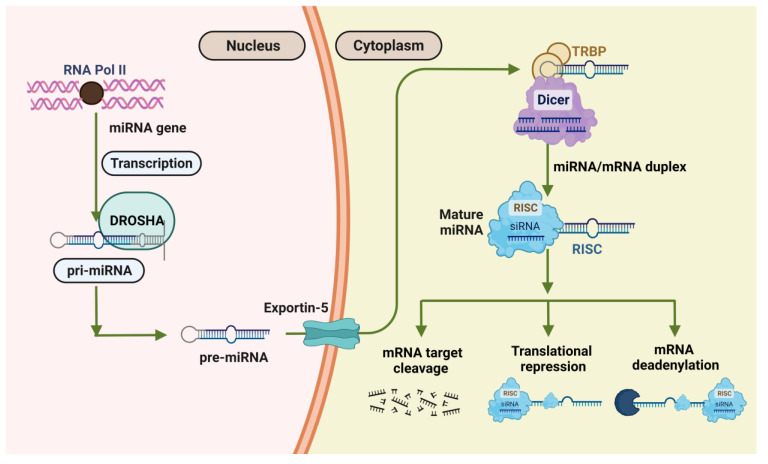
Diagrammatic representation of the events that occur during miRNA biogenesis.

The degree of complementarity between the effector miRNA and the target mRNA influences the process of miRNA-mediated gene silencing. The effector miRNA directs miRISC to the target mRNA. In summary, there are three stages to miRNA biogenesis. The first occurs in the nucleus due to the nuclear RNase III enzyme Drosha cleaving an 80 nt primary or primiRNA translated from the genome [116]. Pre-miRNA is created. As a result, a 60–70 nucleotide stem-loop intermediate is actively transported into the cytoplasm by Ran–GTP and Exportin–5. The RNase III endonuclease Dicer complex breaks down pre-miRNA in the cytoplasm, producing a 20–22 nucleotide double-stranded fragment incorporated into the RNA-induced silencing complex (RISC). The 3’ UTR on target messenger RNA (mRNA) transcripts is where the five regions of miRNAs bind. Frequently, this results in translational inhibition or mRNA destruction (Figure 5) [117].

The precise quantification of the expression of circulating miRNAs is crucial for biological research and early clinical diagnosis. High sensitivity and accuracy are necessary for detecting miRNA targets. Since miRNAs have a small size, short survival time, similar member sequences, and low abundance enrichment in human body fluids, quantitative analysis of miRNAs is a challenge [118]. Conventional detection strategies for miRNA, including Northern blotting, quantitative real-time reverse-transcription (qRT-PCR), miRNA microarrays, fluorescence in situ hybridization, deep sequencing of the transcriptome (RNAseq), and cloning, are basically and widely used technologies [119]. 

### 5.2. Detection Strategies for MiRNAs

#### 5.2.1. Northern Blotting

A well-known miRNA detection technique is Northern blotting (NB), which was used in the initial identification of a miRNA. It can be used to detect miRNA precursors in addition to mature miRNAs. The earliest attempt to methodically measure the expression profile of miRNAs was Northern blotting. It is widely used to visualize the expression of miRNAs of all lengths, from the long primordial miRNA to the mature form [120]. The method involves transferring the extracted RNA to a blotting membrane after first separating it by size using electrophoresis. Following the separation of the RNAs, the membrane is fixed, and sequences of interest are labeled by combining complementary DNA probes. Finally, hybridization with labeled, sequence-specific oligonucleotide probes is used to detect or quantify mature miRNAs. Once the free search has been washed, miRNAs can be identified using autoradiography or other appropriate methods [121]. The RNA was often crosslinked to the blotting membrane to increase the sensitivity of Northern blots. To crosslink RNAs to the nylon membrane, 1-ethyl-3-(3-dimethyl aminopropyl) carbodiimide (EDC) was used as an alternative, leading to a 25–50-fold increase in the sensitivity of miRNA detection. NB analysis has the advantage of implementing a multiplex miRNA detection method using color-coded detection probes, simultaneously detecting mature miRNAs and miRNA precursors [122].

#### 5.2.2. Quantitative Real-Time Reverse-Transcription PCR

A wide dynamic range is typically covered by quantitative real-time reverse transcription (qRT-PCR), which is frequently referred to as the gemstone for measuring gene expression. Reverse transcription of miRNA to cDNA and real-time qPCR monitoring of reaction product accumulation are the two main components of qRT-PCR-based circulating miRNA profiling. Target miRNAs could be reverse transcribed using either general or specific RT primers during the cDNA synthesis step for exact miRNA quantification [123]. Fluorescence is used to monitor the amplification in real time either by using fluorescence probes or a dye that is specific to double-stranded DNA (such as SYBR Green I). Since qRT-PCR techniques have been developed, high-sensitivity miRNA detection has been reduced to a few nanograms of total RNA. The poly(A) polymerase method is more appropriate for detecting multiple miRNAs from a minimal amount of starting material, such as plasma. The "gold standard" for miRNA detection is the TaqMan miRNA assay using stem-loop RT primers and miRNA-specific TaqMan probes [124]. It is well known that RT-qPCR is an effective method for finding miRNAs. However, this method is time-consuming, complex, and expensive thermal cycling equipment is required for amplification and quantification, and it is not appropriate for POC testing [125].

#### 5.2.3. MiRNAs Microarray Technology

The most popular technique for the quick and thorough detection of miRNAs is the microarray. The specific receptors of each target are spatially separated using this detection method to enable multiplexing. Nucleic acid hybridization between target molecules and their complementary probes is the basis for microarrays [126]. Planar arrays and suspension (or on-particle) array are the two basic groups into which these techniques could be separated. Every particle in a suspension array is assigned a matching “barcode” that identifies the analyte it is intended to detect and is attached to capture probes for that analyte. Given the variety of accessible particles, many barcoding techniques, including size, graphic, and often fluorescence labeling, were used. Planar arrays, also known as flat arrays, specifically target catch probes on a flat plane, with each spot focusing on a distinct analyte, using separation as a multiplexing method. Both strategies have intriguing qualities, particularly when it comes to multiplexing [127].

#### 5.2.4. Next-Generation Sequencing

NGS has become a crucial tool in cancer research for identifying microRNAs (miRNAs) and understanding their role in the disease. This technology allows for creating a comprehensive map of miRNA expression across different samples and conditions, which can provide insight into the underlying molecular mechanisms of cancer. RNA sequencing (RNA-seq) begins with extracting and purifying RNA from the sample, which is followed by adding adapters to the 5′ and 3′ ends of each RNA strand. Reverse transcription, PCR amplification, and sequencing are the next steps. One method, called the MicroRNA NGS Data Analysis (miND assay), developed by Khamina et al., allows for the determination of absolute concentrations of miRNAs in total RNA samples obtained from plasma and other liquid biopsies, enabling the comparison of data within or across sample types, which can be helpful in the context of liquid biopsy [128]. In addition, NGS also enables the identification of miRNA–mRNA regulatory networks that are altered in cancer, which can provide insight into the underlying molecular mechanisms of the disease. Furthermore, a recent approach that combines bioinformatics and next-generation sequencing technologies to detect cell-free circulating miRNAs has been reported [129]. In addition to these methods, there is an extensive database entitled Mirandola. According to their extracellular form, miRNAs are divided into four groups: miRNA-Ago2, miRNA-exosome, miRNA-high-density lipoprotein, and miRNA-circulating. The database gives users access to a wide range of data, comprising details on the linked disorders, the tissues, the techniques used to extract the miRNAs, and the experiment’s summary [130]. These miRNAs can be used as biomarkers for the early detection, diagnosis, and monitoring of cancer. In 2016, Oxford Nanopore Technologies successfully launched a portable sequencer that utilized a biological nanopore in its design, opening up the potential for a wide range of practical applications for nanopore sensing technology, including liquid biopsy, point-of-care testing, and personalized medicine [131]. Nanopore sensing is a new and emerging technology for the detection of single molecules, such as oligonucleotides, that can be moved through nanochannels of proteins that make up the pore; in this technique, nano-scale holes are embedded in a thin membrane structure to detect potential changes when charged biological molecules more diminutive than the nanopore passes through the hole. It has also shown clear blocking currents at the single-molecule level. Therefore, nanopore technology has the potential to sense and analyze single-molecule amino acids, DNA, RNA, etc. [132]. Many studies have used biological and solid-state nanopores to detect miRNAs in various tissues. For example, Meni et al. demonstrated the potential of this approach by detecting liver-specific miRNAs at microgram levels in rat liver using nanopore technology for the rapid detection of probe-specific miRNAs (miRNA-122a and miRNA-153) [133]. Wang et al. used hemolysin-based nanopore sensors to detect single-molecule miRNAs in plasma samples from lung cancer patients. The sensor generates target-specific signals using programmable oligonucleotide probes to quantify sub-millimolar levels of cancer-associated miRNAs [134]. These methods have potential application value for quantitative miRNA detection, detection of disease markers, and non-invasive early cancer diagnosis. In addition, the nanopore gene sequencing method allows researchers to investigate the mechanisms of miRNA overexpression. Multiplexed detection of let-7 miRNA is also possible through a nanopore. The thelet-7 family of miRNAs plays a vital role in human development and can be used as biomarkers for disorders such as cancer [135].

#### 5.2.5. In Situ Hybridization

**Figure 6:**
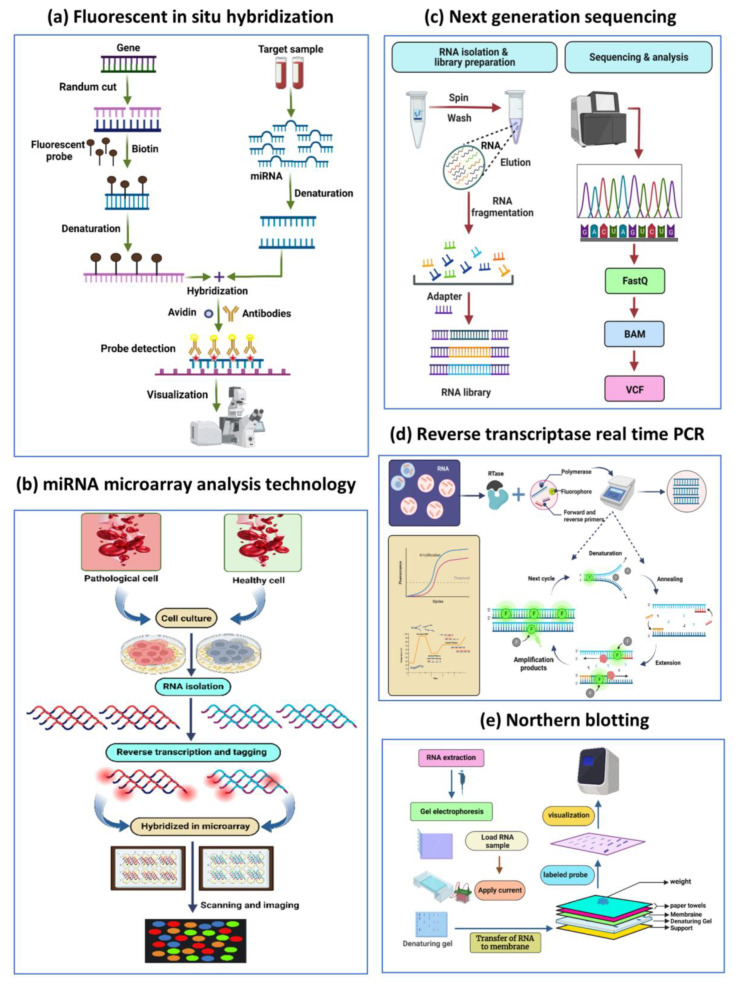
Techniques used for the detection of miRNA. (**a**) Fluorescent in situ hybridization. (**b**) miRNA microarray analysis technology. (**c**) Next-generation sequencing. (**d**) Reverse transcriptase real time PCR. (**e**) Northern blotting.

In situ hybridization (ISH) makes it possible to identify nucleic acid motifs at the molecular level in cell lines. The ISH method identifies the cellular basis of translation and offers details on translation levels in various tissue divisions and cell types for the identification of individual miRNAs and mRNAs. This tissue expression study is essential for understanding the functions of miRNAs in cellular and biochemical functions. This method, first applied to detecting miRNA in 2006, uses labeled complementary nucleic acid probes to find single-stranded DNA or RNA in tissue sections or fixed cells [136]. This technique can now see multiple miRNAs per reaction thanks to the recent development of directly labeled fluorescence probes. The ability to validate these platforms in diagnostic FISH POC settings will be strengthened by additional research into discovering more disease-specific probes and labeling techniques [137]. Even though these methods had sufficient responsiveness, they were time-consuming, needed severe conditions such as high potentials, and demand an expensive equipment and consumables. Therefore, it is critically necessary to create novel techniques for the quick, easy, sensitive, focused, and quantitative detection of miRNAs at low potentials (Figure 6).

## 6. Carbon Nanodots in Biosensing of MiRNAs

Macromolecules and circulating analytes in biological systems must be detected in a way that is efficient, reliable, and inexpensive. Recent developments in the field of biosensors have aided the development of functionalized nanosensors that have the potential to provide a cost-effective, efficient, and quick diagnostic approach for the detection of circulating miRNAs. Along with this, some unique properties—such as biocompatibility, high stability and water dispersibility, and accessible green synthesis, surface functionalization of C-dots that creates a strong interaction between CNDs and biological processes—all these makes them significant for sensing circulating analytes [138]. Fluorescent, colorimetric, chemiluminescent, and surface plasmon resonance are the most common sensing systems used to detect circulating miRNAs [139]. This is due to the relative ease of making fluorescent CNDs and their photostability, which can be used as low-cost alternatives for sensing significant biomarkers (Table 2). Fluorescence-based analytical approaches allow for the accurate, efficient, and reproducible detection of biomarkers and nucleic acids. Furthermore, changes in fluorescent signals caused by biological events such as nucleic acid probe hybridization are detectable. Thus, fluorescence-based detection technologies have become increasingly popular due to these benefits [140].

When a target interacts with a recognition element, a fluorescent biosensor translates information quantitatively or semi-quantitatively. After hybridizing complementary nucleic acid with its target miRNA, fluorescent-based nucleic acid detection can be generally achieved via signal-on (signal production) and signal-off (signal quenching). For DNA hybridization and tumor marker detection, carbon nanomaterial biosensors based on the FRET mechanism have practical utility in research and clinical practice. A FRET sensing platform for sensitive miRNAs detection using the miRNA-155 probe-labeled C-dots as a fluorophore and MnO_2_ nanosheets as a quenching agent was also studied. FRET from modified C-dots to MnO_2_ nanosheets can dramatically reduce the fluorescence of modified C-dots. The quenched fluorescence could be recovered when the target analyte miRNA-155 was introduced [160]. The principal mechanism for sensing miRNA is when the C-dots-miRNA probe is mixed with MnO_2_ nanosheets, which absorb the C-dots-miRNA probe on its surface. Due to FRET, the fluorescence of the C-dots-miRNA probe decreases as the concentration of MnO_2_ nanosheets increases [161]. When the complementary target miRNA was added, specific binding occurred between the search and the target miRNA, causing the C-dots labeled miRNA hybrid to separate from the MnO_2_ nanosheets. As a result, as the concentration of miRNA rises, fluorescence intensity is restored and increased [162]. In recent report using the colorectal cancer-specific miRNA miR-92a-3p as such a target, the efficacy of a ratiometric fluorescence biosensor made of CNDs and acridine orange is evaluated. The variables that determine the viability of the ratiometric fluorescence bioassay are the charges properties of the DNA probe, target miRNA, CNDs, and AO, as well as the fluorescent properties of CDs and AO. The targeted miRNA has a detection limit for the ratiometric fluorescence biosensor: 0.14 nM [143]. CNDs are also used in paper-based analytical devices (PADs), which were previously reported for detecting circular RNA and miRNA-21 from the hippocampal via probe DNA conjugations and in situ manufacture of blue-emissive CNDs. Using miRNA-21 color analysis, stunning blue-to-green and blue-to-red emission color changes of the PADs are obtained [163]. miRNA-21, a predictor of numerous pathologies including cardiovascular illnesses, is detected sensitively and specifically using an easy CNDs-based electron transfer chemiluminescence biosensor [145]. Shandilya et al. designed and constructed a nanophotonic method employing oligonucleotide-conjugated graphene quantum dot–nanoconjugates, which is a derivative of carbon dots for the quick and precise capture of lncRNAs. The technique provides very selective and precise target lncRNA identification. The data also indicated the method’s great practicality and simplicity in determining lncRNAs selectively [164]. Similarly, Chen et al. created a label-free, enzyme-free fluorescent scheme based on strands displacement amplification (SDA) to detect miRNA with extreme sensitivity utilizing CNDs functionalized with sulfydryl (CDs-SH) as the probe. Based on the catalytic oxidation of -SH into -S-S- by hemin/G-quadruplex, CDs-SH demonstrated outstanding response to G-quadruplex DNA against other DNAs [147]. In a study using on CNDs, a sensor for miRNA 9-1 recognition was created. On excellent fluorescence QY, water dissolvable, and low-toxicity CNDs, single-strand DNA with the FAM tag was immobilized. As a physical attribute for sensing, the fluorescent quenching of CNDs caused by the transfer of energy of fluorescence resonance among CNDs and FAM was utilized [155]. Jiang et al. proposed a self-assembled tetrahedral DNA nanostructure coupled with gold nanoparticles (AuNPs) and CNDs. The constructed nanostructure enables double fluorescence channels for the parallel estimation of miRNA and telomerase function, which is also easily be transported within live cells for in situ scanning by adding an iRGD peptide sequence afterwards [160]. Using single-walled carbon nanotubes, a derivative of CNDs that have been sensitized with DNA-CdS QDs, a flexible photoelectrochemical biosensors platform has been created. A practical, accurate, and focused biosensor for the direct detection of miRNAs was developed by integrating with cyclic enzymatic multiplication, offering a unique method for miRNA detection [158]. In another study, they presented the selective and sensitive detection of exosomal miRNAs using a ratiometric fluorescent bio-probe based on DNA-labeled carbon dots (DNA-CNDs) and 5,7-dinitro-2-sulfo-acridone coupling with the target-catalyzing signal amplification, in which high FRET between CNDs and DSA boosted the assay’s sensitivity of the bio probe [165]. Similarly, a new strategy was developed for the construction of a dual-emission fluorescent sensor using a new ratiometric nanohybrid fluorescent probe for the detection of miRNA-21 with dual-colored CNDs (blue CNDs and yellow CNDs) as they are provided with the same excitation wavelength (360 nm), two distinct and steady emission signals (409 and 543 nm) were produced as fluorophores and then their applications for ratiometric miRNA-21 sensing and the bioimaging of cancer cells in a microfluidic device were confirmed [141]. The strong and precise binding of DNA probe functionalized B-CNDs to the complementary miRNA-21 target caused probe structural perturbations and changed fluorescence intensity in both wavelengths as miRNA-21 concentration increased. Thus, because of its rapid reaction, high sensitivity, and technical simplicity, the proposed fluorescent nano-biosensor has become a reliable analytical sensing tool [166]. The great sensitivity (because of CNDs’ high brightness), multiplex capability (due to CNDs’ color tunability), and homogeneous assay formats are all key advantages of CND’s performance in fluorescence biosensors for the detection of circulating nucleic acids [167]. Using CNDs as photosensitizers, TiO_2_ was grown on the edges of gold nanorods (AuNRs) to form dumbbell-shaped structures (AuNRs@end-TiO_2_), which were then hydrophobically attached to fluorine tin oxide (TiO). FTO was bonded to the electrode surface. As a result, a compact photoelectrochemical miRNA-21 was created. Hairpin probes (HPs) were used to bind to the TiO_2_-modified FTO electrode surface, while CNDs-modified homologous DNA (CNDs-cDNA) served as the photosensitive label. When targets were present, the miRNA hybridized with the HP, which caused a double-stranded specific nuclease to associate with the miRNA to the homologous segment of the HP. This released the miRNA, potentially starting a new cycle that would result in signal acquisition [168]. Gold nanoparticles conjugated with CNDs have also demonstrated excellent sensing capability. In a study, gold nanoparticles conjugated with CNDs have also shown excellent sensing capabilities. Photo-assisted biofuel cell-based self-powered biosensors (PBFC-SPBs) are also used in biosensing to identify miRNA. The coupling of PBFC-SPBs for miRNA monitoring with a Cu^2+^/carbon nanotube (Cu^2+^/CNTs) cathode with laccase-mimicking activity made this possible. When the target was identified, the matched miRNA with the same sequence eluted DNA2/CdS from the electrode, resulting in a weak signal. The method does not require the use of an external power source [169]. CNDs have been also widely used for the fluorescent analysis of various targets, including small molecules such as ions, H_2_O_2_, and biomolecules, due to their excellent PL properties. Aptamers were also recognized using CNDs. Aptamers are artificial single-stranded DNA or RNA that have a high affinity for different analytes. Xu et al. created an aptasensor for thrombin detection; it has several aptamer binding sites. Two thrombin aptamers with amino groups were created. They were modified separately on silica nanoparticles and CNDs, and both are capable of recognizing thrombin by forming an intramolecular G-quadruplex [170].

## 7. Conclusions

Non-communicable diseases (NCDs), including cancers, have persisted as major worldwide public health concerns, causing significant death and morbidity. Most molecular processes, including cellular development, differentiation, death, and disarrayed tumorous growth, are controlled by miRNAs, which are crucial in the pathogenesis of cancers. Because of their significant regulatory role, miRNAs as biomarkers are widely employed for early diagnosis of malignant diseases and therapy monitoring. Zillions of miRNAs have different expression patterns and can either be up-regulated or down-regulated in cancer. Therefore, developing reliable and susceptible sensors for miRNA detection and quantification is highly warranted. With their excellent compatibility, distinctive fluorescence spectrum features, and inherent semiconductor properties, CNDs permit the detection of miRNAs by adopting simplified miniature readings. We have primarily focused on three aspects in this review paper. (1) CNDs and their properties—They have gained significant attraction in various domains due to their distinctive physicochemical, optical and electrical properties. In particular, the rich optical and electronic properties of CNDs enable efficient light harvesting, exceptional and tunable photoluminescence (PL), and excellent photoinduced electron transfer. (2) Synthesis and Surface functionalization—CNDs can be synthesized by top–down and bottom–up approaches, and a green synthesis of CNDs is also possible that is novel, facile, and cost-effective. Furthermore, the prepared CNDs will undergo surface functionalization, which conjugates biomolecules with CNDs. (3) Leveraging CNDs for miRNA sensing—Fluorescent biosensors, electrochemiluminescence biosensors, chemiluminescence biosensors, and ratiometric fluorescence are examples of miRNA detection technologies. Liquid biopsy is still in its infancy, but it will provide us with more detailed information on tumor heterogeneity and the development of biosensors for its detection. It is intriguing to think about the future of CNDs-based nano-biosensors for miRNA detection and where they will have the most clinical impact. As we advance in this fourth industrial revolution, the fusion of the physical (hardware), digital (software and artificial intelligence), and biological domains may lead to the creation of point-of-care tests for early cancer diagnosis that are more dependable, sensitive, user-friendly, and affordable.

## Figures and Tables

**Table 2:** List of Various Carbon Nanodots Used in Biosensing of Cell Free Circulating MiRNAs Using Along with Synthesis Sources, Conjugation Chemistry, Analytical Methods, Target miRNAs and Detection Limit.

S. No.	Carbon Nanomaterial	Source and Synthesis	Conjugation Chemistry	Biomolecule (Analyte)	Analytical Method	Detection Limit	Inference	References
1	Carbon nanodots (CNDs)	O-phenylene diamine, 2-amino terephthalic acid by solvothermal method	EDC-NHS	miRNA-21	Fluorescent biosensor	0.03 fM	CNDs are synthesized and conjugated via EDC-NHS chemistry to detect miRNA-21.	[141]
2	PEI-Carbon dots	Polyethyleneimine (PEI) by hydrothermal method	-	miRNA-21	Fluorescence biosensor	-	The synthesized CNDs is employed to detect miRNA-21 by fluorescence biosensor.	[142]
3	CNDs/AO	Citric acid in formamide	π-π conjugation	miRNA-92a-3p	Fluorometric assay (FRET)	0.14 nM	To detect miRNA-92a-3p, CNDs are fabricated and conjugated using π–π conjugation.	[143]
4	CNDs–DNA walker	Citric acid and urea by microwave-assisted method	EDC-NHS	miRNA-21 miRNA-155	Electrochemiluminescence biosensor	33 fM for miRNA-21. 33 aM for miRNA-155	CNDs are created and conjugated via EDC-NHS chemistry to discover miRNA-21 and miRNA-155.	[144]
5	CNDs	Oxidized maple leaf by a pyrolytic method	EDC-NHS	miRNA-21	Electrochemiluminescence biosensor	21 aM	CNDs are synthesized and conjugated via EDC-NHS chemistry to detect miRNA-21 associated with breast cancer.	[140]
6	CNDs	Tiger nut milk by carbonization	-	miRNA-21	Chemiluminescence biosensor	0.721 fM	Synthesized CNDs are used to detect miRNA-21 associated with cardiovascular disease.	[145]
7	CNDs	Glutaraldehyde, nitro benzaldehyde by solvothermal method	-	miRNA-21	Fluorescence sensor	0.03 fM	An miRNA-21 associated with breast cancer is identified using a fluorescence sensor that is based on carbon dots.	[146]
8	CNDs	Malic acid centrifugation	EDC-NHS	miRNAs	Fluorescence	0.03 pM	The synthesized CNDs is conjugated via EDC NHS chemistry and used to detect miRNA.	[147]
9	CNDs	Citric acid by microwave method	π-π stacking	miRNAs	Fluorescence biosensor	2.78 fM	CNDs were synthesized and employed to detect miRNAs by fluorescence biosensor.	[148]
10	CNDs	Tree leaves by hydrothermal method	EDC-NHS	miRNA-155	Fluorescence biosensor FRET	0.3 aM	CNDs were synthesized and conjugated via EDC-NHS chemistry and were used to detect miRNA-155 by fluorescence biosensor.	[149]
11	CNDs/BHQ 2	Ethane diamine, p-benzoquinone	Maleimide-thiol	miRNA-141	FRET	16.5 pM	miRNA-14 is conjugated with synthesized CNDs via maleimide–thiol conjugation chemistry and detected by a fluorimetry test.	[150]
12	Carbon nanotubes (CNTs)	Hydrogen tetrachloroaurate trihydrate	EDC-NHS	miRNA-21	Fluorescence biosensor	36 pM	A synthesized CNT is conjugated via EDC-NHS chemistry to detect intracellularly miRNAs-21.	[151]
13	CNDs	Pyrolysis synthesis	Amine-amine conjugation	miRNA-21	Ratiometric fluorescence	1 pM	Synthesized CNDs were used to detect miRNA-21 associated with gastrointestinal cancer.	[152]
14	CNDs	Citric acid ethylene diamine/carbonization	Amine -glutaraldehyde	miRNA-155	FRET	0.1 aM	Fabricated CNDs are used to identify miRNA-155 present in cancer cells.	[153]
15	CNTs (MWCNT/AuNCs)	Carboxylic acid-ultrasonic cell disruption	Thiol conjugation	miRNA-155	FRET	33.4 fM	CNTs are synthesized and used to detect miRNA-155.	[154]
16	CNDs	Citric acid– hydrothermal	π-π stacking	micro-RNA	Fluorescence		A CNDs is used to detect miRNA by fluorescence method.	[155]
17	CNTs	-		miRNA-21	Electrochemical biosensor	1.95 fM	miRNA-21 is detected by a carbon nanotube-based electrochemical biosensor.	[156]
18s	Carbon nanofibers/SPE	-	Amine-carboxylic acid conjugation	miRNA-34a	Electrochemical biosensor	54 pM	The electrochemical biosensor is utilized to detect miRNA-34a using carbon nanofibers.	[157]
19	DNA-CNDs/CNTs	-	π-π stacking	miRNA-7f	Photoelectrochemical biosensor	34 fM	A CNTs is used to detect miRNA-7f by a photoelectrochemical method.	[158]
20	Carbon nanoparticles/ssDNA probe	Graphite electrode by an electro-oxidation method	π-π stacking	miRNA let-7a	Fluorescence	0.35 pM	Synthesized carbon nano-particles conjugated via π–π stacking are used to detect miRNA let-7a.	[159]

Abbreviations: Forster resonance energy transfer (FRET), Single-stranded DNA (ssDNA), Ethyl(dimethylaminopropyl)carbodiimide/N-hydroxysuccinimide (EDC-NHS), Screen-printed electrodes (SPEs), Black hole quencher 2 (BHQ-2), Multiwalled carbon nanotube (MWCNT), Gold nanocomposites (AuNCs), Polyethyleneimine (PEI), Acridine orange (AO), Femtomolar (fM), Nanomolar (nM), Attomolar (aM), Picomolar (pM).

## Data Availability

Not applicable.

## Abbreviations

AO	Acridine Orange
AuNPs	Gold Nanoparticles
ccfmiRNAs	Circulating Cell-Free Micro Ribonucleic Acids
CdS	Cadmium Sulfide
CdSe	Cadmium Selenide
CNDs	Carbon Nanodots
ECL	Electrochemiluminescence
EDC	1-Ethyl-3-(3-Dimethylaminopropyl) carbodiimide
EYO	Egg Yolk Oil
FRET	Fluorescence Resonance Energy Transfer
GO	Graphene Oxide
miRISC	miRNA-Induced Silencing Complex C
miRNAs	microRNAs
NO_2_	Nitrogen Dioxide
PEC	Photoelectrochemical
PEG	Polyethylene Glycol
PL	Photoluminescence
PM	Particulate Matter
QDs	Quantum Dots
qRT-PCR	Quantitative Real-Time Polymerase Chain Reaction
QY	Quantum Yield
RCG	Raw Cashew Gum
RT	Room Temperature
SPDP	N succinimidyl-3-(2-pyridyldithio) propionate
